# Identification and Characterization of Human Observational Studies in Nutritional Epidemiology on Gut Microbiomics for Joint Data Analysis

**DOI:** 10.3390/nu13093292

**Published:** 2021-09-21

**Authors:** Mariona Pinart, Katharina Nimptsch, Sofia K. Forslund, Kristina Schlicht, Miguel Gueimonde, Patrizia Brigidi, Silvia Turroni, Wolfgang Ahrens, Antje Hebestreit, Maike Wolters, Andreas Dötsch, Ute Nöthlings, Kolade Oluwagbemigun, Rafael R. C. Cuadrat, Matthias B. Schulze, Marie Standl, Michael Schloter, Maria De Angelis, Patricia Iozzo, Maria Angela Guzzardi, Geertrui Vlaemynck, John Penders, Daisy M. A. E. Jonkers, Maya Stemmer, Giulia Chiesa, Duccio Cavalieri, Carlotta De Filippo, Danilo Ercolini, Francesca De Filippis, David Ribet, Najate Achamrah, Marie-Pierre Tavolacci, Pierre Déchelotte, Jildau Bouwman, Matthias Laudes, Tobias Pischon

**Affiliations:** 1 Molecular Epidemiology Research Group, Max Delbrück Center for Molecular Medicine in the Helmholtz Association (MDC), 13125 Berlin, Germany; Mariona.PinartGilberga@mdc-berlin.de (M.P.); tobias.pischon@mdc-berlin.de (T.P.); 2 Experimental and Clinical Research Center, A Cooperation of Charité-Universitätsmedizin Berlin and Max Delbrück Center for Molecular Medicine, Lindenberger Weg 80, 13125 Berlin, Germany; Sofia.Forslund@mdc-berlin.de; 3 Charité-Universitätsmedizin Berlin, Corporate Member of Freie Universität Berlin, Humboldt-Universität zu Berlin, Berlin Institute of Health, 10117 Berlin, Germany; 4 Host-Microbiome Factors in Cardiovascular Disease Lab, Max Delbrück Center for Molecular Medicine in the Helmholtz Association (MDC), 13125 Berlin, Germany; 5 German Centre for Cardiovascular Research (DZHK), Partner Site Berlin, 10785 Berlin, Germany; 6 Berlin Institute of Health (BIH), 10178 Berlin, Germany; 7 Structural and Computational Biology Unit, European Molecular Biology Laboratory, 69117 Heidelberg, Germany; 8 Institute of Diabetes and Clinical Metabolic Research, University of Kiel, 24105 Kiel, Germany; Kristina.Schlicht@uksh.de (K.S.); matthias.laudes@uksh.de (M.L.); 9 Department of Microbiology and Biochemistry of Dairy Products, IPLA-CSIC, 33300 Villaviciosa, Spain; mgueimonde@ipla.csic.es; 10 Diet, Microbiota and Health Group, Instituto de Investigación Sanitaria del Principado de Asturias (ISPA), 33011 Oviedo, Spain; 11 Department of Medical and Surgical Sciences, University of Bologna, Via Massarenti 9, 40138 Bologna, Italy; patrizia.brigidi@unibo.it; 12 Department of Pharmacy and Biotechnology, University of Bologna, Via Belmeloro 6, 40126 Bologna, Italy; silvia.turroni@unibo.it; 13 Leibniz Institute for Prevention Research and Epidemiology-BIPS, 28359 Bremen, Germany; ahrens@leibniz-bips.de (W.A.); hebestr@leibniz-bips.de (A.H.); wolters@leibniz-bips.de (M.W.); 14 Institute of Statistics, Bremen University, 28359 Bremen, Germany; 15 Department of Physiology and Biochemistry of Nutrition, Max Rubner-Institut (MRI)-Federal Research Institute of Nutrition and Food, 76131 Karlsruhe, Germany; andreas.doetsch@mri.bund.de; 16 Nutritional Epidemiology Unit, Institute of Nutrition and Food Sciences, University of Bonn, 53115 Bonn, Germany; u.noethlings@uni-bonn.de (U.N.); koluwagb@uni-bonn.de (K.O.); 17 Department of Molecular Epidemiology, German Institute of Human Nutrition Potsdam-Rehbruecke, 14558 Nuthetal, Germany; Rafael.Cuadrat@dife.de (R.R.C.C.); mschulze@dife.de (M.B.S.); 18 Institute of Nutritional Science, University of Potsdam, 14558 Potsdam, Germany; 19 German Center for Diabetes Research (DZD), 85764 Neuherberg, Germany; 20 Institute of Epidemiology, Helmholtz Zentrum München-German Research Center for Environmental Health, 85764 Neuherberg, Germany; marie.standl@helmholtz-muenchen.de; 21 Research Unit for Comparative Microbiome Analysis, Helmholtz Zentrum München-German Research Center for Environmental Health, 85764 Neuherberg, Germany; schloter@helmholtz-muenchen.de; 22 Department of Soil, Plant and Food Sciences, University of Bari Aldo Moro, 70126 Bari, Italy; maria.deangelis@uniba.it; 23 Institute of Clinical Physiology, National Research Council, Via Moruzzi 1, 56124 Pisa, Italy; patriciaiozz@ifc.cnr.it (P.I.); m.guzzardi@ifc.cnr.it (M.A.G.); 24 Department Technology and Food, Flanders Research Institute for Agriculture, Fisheries and Food, 9090 Melle, Belgium; geertrui.vlaemynck@ilvo.vlaanderen.be; 25 Department of Medical Microbiology, School of Nutrition and Translational Research in Metabolism (NUTRIM) and Care and Public Health Research Institute (CAPHRI), Maastricht University Medical Center, 6200 MD Maastricht, The Netherlands; j.penders@maastrichtuniversity.nl; 26 Department of Internal Medicine, Division Gastroenterology-Hepatology, NUTRIM School of Nutrition and Translational Research in Metabolism, Maastricht University Medical Center, 6200 MD Maastricht, The Netherlands; d.jonkers@maastrichtuniversity.nl; 27 Department of Industrial Engineering and Management, Ben-Gurion University of the Negev, Beer-Sheva P.O. Box 653, Israel; mayast@post.bgu.ac.il; 28 Department of Pharmacological and Biomolecular Sciences, Università degli Studi di Milano, 20133 Milan, Italy; giulia.chiesa@unimi.it; 29 Department of Biology, University of Florence, Via Madonna del Piano 6, 50019 Florence, Italy; duccio.cavalieri@unifi.it; 30 Institute of Agricultural Biology and Biotechnology National Research Council, Via Moruzzi 1, 56124 Pisa, Italy; carlotta.defilippo@ibba.cnr.it; 31 Department of Agricultural Sciences, University of Naples Federico II, 80055 Portici, Italy; danilo.ercolini@unina.it (D.E.); francesca.defilippis@unina.it (F.D.F.); 32 Task Force on Microbiome Studies, University of Naples Federico II, 80134 Naples, Italy; 33 INSERM UMR 1073 “Nutrition, Inflammation and Gut-Brain Axis Dysfunctions”, UNIROUEN, Normandie University, 76000 Rouen, France; david.ribet@inserm.fr (D.R.); najate.achamrah@univ-rouen.fr (N.A.); mp.tavolacci@chu-rouen.fr (M.-P.T.); Pierre.Dechelotte@chu-rouen.fr (P.D.); 34 Department of Nutrition, CHU Rouen, 76000 Rouen, France; 35 INSERM CIC-CRB 1404, CHU Rouen, 76000 Rouen, France; 36 Microbiology and Systems Biology Group, TNO, Utrechtseweg 48, 3704 HE Zeist, The Netherlands; jildau.bouwman@tno.nl; 37 Biobank Technology Platform, Max Delbrück Center for Molecular Medicine in the Helmholtz Association (MDC), 13125 Berlin, Germany; 38 Biobank Core Facility, Berlin Institute of Health at Charité-Universitätsmedizin Berlin, 10178 Berlin, Germany

**Keywords:** microbiome, dietary intake, metadata, data integration, data sharing, observational studies, metabolome

## Abstract

In any research field, data access and data integration are major challenges that even large, well-established consortia face. Although data sharing initiatives are increasing, joint data analyses on nutrition and microbiomics in health and disease are still scarce. We aimed to identify observational studies with data on nutrition and gut microbiome composition from the Intestinal Microbiomics (INTIMIC) Knowledge Platform following the findable, accessible, interoperable, and reusable (FAIR) principles. An adapted template from the European Nutritional Phenotype Assessment and Data Sharing Initiative (ENPADASI) consortium was used to collect microbiome-specific information and other related factors. In total, 23 studies (17 longitudinal and 6 cross-sectional) were identified from Italy (7), Germany (6), Netherlands (3), Spain (2), Belgium (1), and France (1) or multiple countries (3). Of these, 21 studies collected information on both dietary intake (24 h dietary recall, food frequency questionnaire (FFQ), or Food Records) and gut microbiome. All studies collected stool samples. The most often used sequencing platform was Illumina MiSeq, and the preferred hypervariable regions of the 16S rRNA gene were V3–V4 or V4. The combination of datasets will allow for sufficiently powered investigations to increase the knowledge and understanding of the relationship between food and gut microbiome in health and disease.

## 1. Introduction

Microbiome research has gained popularity over the past 15 years thanks to a reduction in the cost of high-throughput sequencing technology and the increased availability of sophisticated mathematical and computational techniques, allowing for a better quantification of the microbial composition and, therefore, an understanding of its impacts on human health [1,2]. Firmicutes and Bacteroidetes are the two dominant phyla. Together, Firmicutes and Bacteroidetes represent approximately 90% of the total community of the adult human gut microbiota. Other subdominant phyla, namely Proteobacteria, Actinobacteria, and Verrucomicrobia, are also present [3,4]. The primary determinants capable of modulating the gut microbial composition are diet and interindividual variations, such as epigenetic variations of the host [2]. A diverse diet leads to a diverse microbiome, which, in turn, is considered a healthy microbiome [5]. However, intra- and interindividual variations, as well as the temporal dynamics of the microbiome, preclude a single narrow definition of a healthy human gut microbiome [6].

Previous research projects such as the National Institutes of Health (NIH) Human Microbiome Project (HMP) [7] in the USA (2007–2014) and the Metagenomics of The Human Intestinal Tract (MetaHIT) [8] in Europe (2008–2012) have applied advanced sequencing and bioinformatics tools. These projects have undertaken impactful research on the influence of diet and nutrition on the gut microbiota in health and diseases, including inflammatory bowel disease (IBD) and cardiometabolic diseases. The term “microbiome” can be defined—according to a consensus definition recently suggested by Berg et al. [9]—as “a characteristic microbial community occupying a reasonable well-defined habitat which has distinct physio-chemical properties”, which may help to improve the standardization of microbiome studies in the future. The term “metagenome” refers to the collective genomic content from the members of a microbiota [6]. For conditions such as obesity, some studies using high-throughput sequencing technology have suggested that obese individuals have a higher proportion of Firmicutes and a lower proportion of Bacteroidetes compared to lean people, although the results from various studies have not been consistent [10]. Other studies have shown that obesity is associated with lower proportions of the family *Rikenellaceae* (phylum Bacteroidetes) and the genus *Oscillospira* (phylum Firmicutes, family *Ruminococcaceae*), and with higher proportions of genera *Blautia* and *Roseburia* (phylum Firmicutes) [11]. Whereas *Akkermansia muciniphila* (phylum Verrucomicrobia) has been consistently linked to improved metabolic health and leanness [12], *Collinsella* (phylum Actinobacteria) has been associated with obesity [3]. However, the results from different studies vary substantially, and to date, no consistent taxonomic signature of obesity has been identified in the human gut microbiome [13,14]. A meta-analysis showed much more consistent reports of taxonomic shifts in IBD than those observed for obesity, including a depletion of Firmicutes and Bacteroidetes and enrichment in Proteobacteria and Actinobacteria, although no individual microbes were consistently associated with IBD across studies [15]. Further, it is unclear to what extent differences in diets (or dietary behavior) account for differences in the microbiome between obese and non-obese individuals. For example, it is known that high-fiber diets influence the human microbiome and immune system in healthy adults [16,17], and that obese individuals consume less fiber as compared to non-obese individuals [18,19,20]. However, one study found sex differences regarding fiber intake, which could be possibly explained by the fact that women tend to be more conscious about their health and better informed about food and nutrition compared to their male counterparts [19].

Overall, inconsistent results from individual studies limit our current understanding of the exact associations between the human gut microbiome and disease [21], which could be due to selection bias, geographic differences, unknown confounding factors, differences on taxonomical resolution, or a lack of standard sampling collection, processing, and analysis methods, among other factors [22]. The Knowledge Platform (KP) on Food, Diet, Intestinal Microbiomics and Human Health (KP-INTIMIC) consortium (2019–2021) (https://dashin.eu/jpi-kp/pages/home/, accessed on 10 July 2021) aims to foster transnational and multidisciplinary collaboration and networking to accelerate, further develop, and increase the impact of gut microbiome research related to human health. The KP-INTIMIC further aims to develop strategies to standardize and harmonize datasets to minimize bias in data analysis, provide the necessary infrastructure for data sharing and data integration, and conduct use cases using data from animal and human studies identified within the consortium. The use cases will also help to move from association to causality between gut microbiome and disease.

Here, we present a description of the design, characteristics, methods, and available data and metadata of epidemiological observational studies on food, diet, and gut microbiomics identified within the context of KP-INTIMIC. As in a previous JPI Knowledge Hub, the European Nutritional Phenotype Assessment and Data Sharing Initiative (ENPADASI) [23,24], the identified studies may be used in future federated individual-level joint data analyses examining the human gut microbiome and the role of diet in health and disease in the areas of early infancy, the development of sub-chronic and chronic diseases during lifespan, and aging. “Federated” refers to the joint analyses of individual-level data using the statistical platform DataSHIELD, a flexible, modular, free, open-source solution that allows the conduct of analysis without the need of individual studies to physically transfer their data into a single central database, as a way to circumvent privacy, ethical, and legal constrains of observational studies that often preclude the physical sharing of data [25].

## 2. Materials and Methods

### 2.1. Consortium Assembly

KP-INTIMIC is a knowledge hub comprising 55 partners from 9 countries with the aim of fostering studies on the microbiome, nutrition, and health, making them findable, accessible, interoperable, and reusable (FAIR) to the scientific community to reduce fragmentation. The consortium also aims to (1) standardize and harmonize data for comparability, (2) move from association to causality, and (3) facilitate data sharing [25,26]. The consortium was assembled in response to a call by the Joint Programming Initiative “A Healthy Diet for a Healthy Life” (JPI-HDHL) ERA-Net Cofund “Interrelation of the INtesTInal MICrobiome, Diet and Health” (HDHL-INTIMIC). The research groups that showed an interest in the call submitted an “Expression of Interest” letter to the Call Secretariat. Subsequently, the research groups networked to frame the KP-INTIMIC program proposal, in which an initial list of 36 human studies (17 observational and 19 intervention) and 11 model organism studies potentially available within the consortium were provided.

### 2.2. Steps to Develop a Template for Study Metadata Collection

We developed a template to gather meta-information from each observational study identified in the KP-INTIMIC network using an already existing template from the previous JPI European Nutritional Phenotype Assessment and Data Sharing Initiative (ENPADASI) [23]. The template was adapted for the collection of microbiome-specific metadata. A three-step approach was followed to develop a template suitable for the KP-INTIMIC needs. The first step was to agree with our partners on the type of information we would like to collect from the identified studies. To this end, a virtual Working Group meeting was organized, and discussions were held regarding types of metadata. The second step was to seek advice from an expert in computational science to discuss relevant microbiome metadata that would be interesting to collect for the use cases. The third step was to hold a meeting with the partners responsible for the creation of the templates (human (intervention and observational) and animal studies) to further discuss the templates for metadata collection of human and animal studies, as well as the strategies to harmonize the metadata related to the microbiome. To capture all relevant observational studies, human studies of various designs (cohort, case-control, or cross-sectional) were collected. However, in the present report, we only describe studies that collected data on nutrition (e.g., dietary assessment), as well as stool samples.

Most of the information included in the template was taken from the template of a previous JPI-HDHL call [23]. Briefly, the following metadata were obtained from the studies: (1) general study information (study name, link to study website, funding body, coordinating center), (2) scope of the study, (3) study design and recruitment, (4) exposure measurements (dietary intake, alcohol and tobacco consumption, physical activity, sedentary behavior, anthropometric measurements, sociodemographic information, and health status), (5) main health-related outcome, and (6) laboratory measurements in biological samples (traditional biomarkers, omics biomarkers, e.g., proteomics, genomics, and microbiomics). Furthermore, the template also aimed to collect information on signed informed consent, ethics committee approval, and whether the data owners wanted to share the raw data and/or metadata within and outside the KP-INTIMIC consortium.

We first circulated the template among the principal investigators (PIs) from the 17 observational studies listed in the KP-INTIMIC program proposal for the confirmation of participation. Then, we circulated the template among all KP-INTIMIC partners to identify more studies. The PIs filled in the template and sent it back with the requested meta-information to the Max Delbrück Center for Molecular Medicine, where they were kept and aggregated into a single meta-database for integration in KP-INTIMIC. An initial list of 27 studies was obtained, but 4 were finally excluded, resulting in 23 studies. Reasons for exclusion were as follows: intervention design (*n* = 2), no collection of data on dietary intake, and no collection of stool samples for gut microbiome measurement (*n* = 2).

## 3. Results

Within the KP-INTIMIC, we identified 23 observational studies conducted in Italy (*n* = 7), Germany (*n* = 6), the Netherlands (*n* = 3), Spain (*n* = 2), Belgium (*n* = 1), and France (*n* = 1), and multinational studies conducted in two or more countries (including Burkina Faso, Cyprus, Denmark, Estonia, France, Germany, Hungary, Italy, and Sweden) (*n* = 3) (Table 1). Sixteen studies were population-based, whereas seven were disease-based (eating disorders; Parkinson’s disease; chronic kidney diseases; cardiometabolic diseases, including IBD (including ulcerative colitis and Crohn’s disease); and irritable bowel syndrome). Of the 16 population-based studies, 13 were of cohort design and 3 were of cross-sectional design. Of the seven disease-based studies, four were of cohort design and three were of cross-sectional design. Of the seven disease-based studies, one had no controls and exclusively recruited diseased individuals with chronic kidney disease (Medika, Italy), whereas the other six primarily recruited disease-based participants but also included a non-diseased control group, which was mainly recruited through advertisements. Of the 23 studies, 8 studies recruited children, of which 3 (birth cohort) studies recruited children at birth (LISA, Germany; LucKI Gut Study, Netherlands; DORIAN-PISAC, Italy), 3 in the first year of life (Infant microbiome studies, Italy; EarlyMicroHealth, Spain; DONALD study, Germany), and 2 studies recruited children and adolescents (IDEFICS/I.Family cohort, TransMic, Multinational). The other 15 studies recruited adult populations. Six studies were still ongoing. Of the 23 observational studies, 15 studies recruited <1000 individuals, 4 recruited ≥1000 individuals (FoCus, and LISA, Germany; Metacardis, Multinational; IBD South Limburg Cohort, Netherlands), and 3 recruited over 10,000 individuals (DONALD, EPIC-Potsdam, IDEFICS/I.Family cohort, and NAKO, Germany), although data on the gut microbiome or collected stool samples were often available only in subsamples.

### 3.1. Assessment of Dietary Intake and Covariates

Twenty-two studies collected data on dietary intake, whereas one did not (Effect of food on gut microbiota and metabolites in Parkinson patients, Belgium). Seventeen studies collected dietary intake using a single method, such as the food frequency questionnaire (FFQ) (*n* = 9), food records (*n* = 4), and other forms (*n* = 4). Four studies collected information using two methods (24 h dietary recall and FFQs), and none used all three methods (24 h dietary recall, FFQs and food records) (Table 2). Multiple 24 h dietary recalls were administered in five studies. FFQs were semiquantitative (*n* = 13) and qualitative (*n* = 1).

Information on further collected data, such as alcohol and tobacco consumption, physical activity, anthropometry, socioeconomic status (SES) (including sex, age, residence, country of birth, country of citizenship, marital status, income, and education level, among other variables), medication use, and health status are described in Supplement Table S1.

### 3.2. Biological Samples and Microbiome Measurements

All the studies collected stool samples, and all but one study (NAKO, Germany) already had microbiome data available from the stool samples. Three studies also collected saliva (NAKO, Germany; Diet4MicroGut, Italy) or oral swabs (Infant microbiome studies, Italy) for the microbiome, although data on the microbiome was only available in the two Italian studies (Table 3).

The preferred sequencing platform was the Illumina MiSeq (*n* = 18), and the preferred method was amplicon sequencing of the 16S rRNA gene (variable regions V3–V4 or V4). Seven studies conducted shotgun metagenome sequencing (Elderly microbiome studies (centenarians and semi-supercentenarians), Diet4MicroGut, Italy; MetaCardis, TransMic, Multinational; IBD South Limburg cohort, Maastricht IBS Study, The Netherlands; EarlyMicroHealth, Spain). The microbiome sequencing platforms used in shotgun metagenomic studies were the Illumina HiSeq (*n* = 2), Illumina NextSeq (*n* = 2), Illumina NovaSeq (*n* = 1), Illumina MiSeq (*n* = 1) and Ion-Proton (*n* = 1). Only two studies (IDEFICS/I.Family cohort, TransMic, Multinational) collected information on the type of meal last eaten prior to sample extraction (Table 4).

Information on biomarkers, such as blood lipids, glucose/insulin, inflammatory markers, adiposity biomarkers, and metabolome data, are described in Supplement Table S2. In total, 14 studies had data on blood lipids (HDL, LDL, and total cholesterol) and glucose/insulin (glucose, HbA1c, fasting insulin, C-peptide), 16 studies had data on inflammatory biomarkers (C-Reactive Protein (CRP), interleukin 6 (IL-6), tumor necrosis factor (TNF)-α), and 10 studies had data on adiposity biomarkers (adiponectin, leptin). Moreover, 15 studies collected metabolome data from stool, urine, plasma, or serum. These studies mainly used nuclear magnetic resonance (NMR) and mass spectrometry (MS) in different forms, such as gas chromatography/mass spectrometry (GC/MS) and liquid chromatography/tandem mass spectrometry (LC/MS/MS).

### 3.3. Informed Consent, Ethics and Data Sharing

All the studies were approved by an Ethics Committee and had collected the informed consent from the individuals participating in their study. In total, 12 studies showed interest in the storage and data sharing within the KP-INTIMIC consortium, although some of them needed confirmation or requested an agreement form. Storing and sharing raw data directly within KP-INTIMIC is restricted by study agreements or consent, necessitating a federated approach similar to the one proposed here for fully exploring their potential. All studies expressed their will to store and share raw data for specific research projects outside the INTIMIC, with the exception of three studies (Effect of food on gut microbiota and metabolites in Parkinson patients, Belgium; EPIC-Potsdam, Germany, TransMic, Multinational). Regarding metadata, all but the Belgian study agreed to share metadata, although some of them needed confirmation or required board approval (Supplement Table S3).

## 4. Discussion

Within the KP-INTIMIC, we identified a total of 23 observational studies which were mainly of longitudinal or cross-sectional design. These studies were conducted in 12 European countries and 1 African country, with data on dietary intake, microbiome, and/or health outcomes. Sixteen studies recruited individuals from the general population whilst seven were disease-based. Most of the studies included adults. Twenty-one studies had data both on dietary assessment and the gut microbiome, whereas one study had data on dietary intake but not on the gut microbiome, although stool samples were collected. Another study had data on the microbiome but not on dietary intake. The studies mostly amplified and sequenced hypervariable regions of the 16S rRNA gene, such as the V3 and V4, using the Illumina MiSeq sequencing platform. Seven studies conducted shotgun metagenome sequencing mostly using the Illumina (MiSeq, NextSeq, NovaSeq, or HiSeq) sequencing platform, and fifteen studies had also data on the urine, stool, plasma, or serum metabolome.

Whereas observational studies cannot prove causality, they have the advantage of recruiting individuals from the general population. Therefore, their results are more generalizable. In addition, observational studies are usually able to assess habitual dietary intake in relation to the gut microbiome. Conversely, intervention studies may select specific population groups (e.g., obese only), which can hinder the application of their results to the general population. Further, dietary intervention studies usually focus on specific short-term multimodal interventions, which often limit interpretability and generalizability. The impact of short-term dietary changes on the gut microbiome is also transient. Of the 16 (70%) population-based studies of the KP-INTIMIC, 14 recruited individuals from the general population, and 2 recruited individuals from a convenience sample. Of the disease-based studies, individuals were recruited from primary, secondary, or tertiary healthcare settings. In addition, 17 studies (74%) were of longitudinal design, which may have allowed the researchers to examine the temporal relationships of the gut microbiota, as well as the dynamic nature of those factors that may modulate the gut microbiota composition, such as dietary intake [22]. Though the identified studies in the KP-INTIMIC used various types of dietary assessment methodologies, successful federated joint meta-analysis can still be conducted, as shown previously [24]. Another advantage of conducting joint data analysis using data from the identified studies is related to the increase in the statistical power [13]. Sufficiently powered studies may find more consistent dysbiosis–disease associations, and may be able to identify taxonomic signatures that may help define and characterize the composition of a dysbiotic gut microbiome [22]. Moreover, larger and representative sample populations of healthy individuals covering different ages and cultures (races or ethnicities) offer an opportunity to examine how the dynamics of gut microbiomes shift during the lifespan, vary between populations, and respond to lifestyle changes [47].

Divergences between individual microbiome studies with similar designs may be explained, among others, by technical factors, which may lead to systematic or nonsystematic errors. In turn, this may lead to misclassification, which can be differential (e.g., systematic, which would be a bias) or nondifferential (e.g., random, which would not be a bias). Technical factors cover stool sampling and preservation methods, as well as DNA isolation and extraction. These aspects are interrelated, since it is thought that both sample collection and preservation methods may have a profound effect on the output of high-throughput sequencing-based technologies used for microbiome determination [22,48]. For example, a cohort study, which quantified technical factors during sample preparation that may affect the characterization of the gut microbiome, showed that DNA extraction methods had the highest impact on the observed microbiome variability, precluding a clear picture of the microbiome signatures of various health and nutrition factors [48]. Research in food, diet and the gut microbiome would largely benefit from initiatives joining efforts to propose guidelines to standardize stool collection and preservation methods. In this regard, the KP-INTIMIC provides an overview on relevant initiatives to develop a collection of standard operating procedures (SOPs) tools for the standardization of wet lab procedures, and data integration of different omics technologies. It has been shown that different storage conditions have significant effects on the health status indicators of the microbiota, such as its richness (quantity of microbes) and its biodiversity (quantity of species), measured by means of alpha diversity (Shannon Diversity index (SDI)), beta diversity measurements (e.g., UniFrac, Bray-Curtis dissimilarity (BC)), and taxa abundances in comparison to immediate freezing (at −20 °C or −80 °C) [22]. By conducting federated joint data analyses using studies from the KP-INTIMIC consortium, we will gain insights on whether study-specific differences in technical factors play a role in the studied associations. 

Traditionally, gut microbiota modulation has been characterized using culture-dependent techniques, which have failed to culture the majority of the microbial ecosystem [6]. Nowadays, high-throughput techniques such as the 16S or shotgun metagenomics (the so-called next generation sequencing techniques) are increasingly used to study the microbiome to identify the genetic material of the microbes [2]. Therefore, results from studies using culture-dependent techniques cannot be easily compared with those using culture-independent techniques. Next-generation sequencing approaches include metagenome sequencing and marker gene sequencing [47]. Whereas the former focuses on sequencing all of the microbial genes from a given sample to provide a more refined and comprehensive knowledge of the composition and genetics of the gut microbiota, the latter focuses on sequencing one or more specific gene regions, such as the 16S rRNA gene, specific to the bacteria and archaea of taxonomic relevance, providing a broad picture of the types of microbes present in the gut [2]. Thus, the two approaches show both advantages and disadvantages. Because metagenome sequencing generates larger volumes of data, it requires more computationally intensive analysis compared to marker gene sequencing, which increases the overall cost dramatically together with an increased sequencing cost and an increase in working hours. However, though cheaper, one of the main caveats of marker gene sequencing is its limited resolution when it comes to the identification of microbial taxa since it only depicts the relative abundance of targeted organisms [2]. Moreover, marker gene sequencing is highly dependent on the primers used for region amplification, which can lead to bias if the selected primers lack sensitivity to certain microorganisms. Studies using marker gene sequencing can only be comparable if the same region is amplified. In addition, marker gene sequencing is not the best approach to identify low abundant and rare microbial groups [6]. Despite these limitations, our collection of studies enables comparisons since multiple studies amplified the same region, e.g., V4 (*n* = 3 studies) and V3–V4 (*n* = 11), and five have conducted both marker gene and shotgun metagenome sequencing. This underlines the importance of standardizing, providing detailed descriptions of methods, and determining technical factors that are critical for the comparability of studies [48].

Another relevant aspect that needs to be addressed to advance the microbiome field is the examination of the functional products of the microbes by means of metabolomics and the underutilized meta-transcriptomics technology approach [49]. Of the 23 studies, 15 identified in the KP-INTIMIC have also collected data on metabolomics using different strategies such as the targeted and untargeted metabolomics in urine, stool, plasma, or serum. The metabolomics investigations in microbiome studies are fundamental in assessing the role of diet in shaping the functions of the gut microbiome. In fact, many health-related microbial metabolites, such as short-chain fatty acids, TMAO, urolithins, and bile acids, have dietary nutrients as precursors. Targeted metabolomics is a strategy characterized by a predefined list of molecules, which can be detected and quantified with high-quality standards. A caveat is that targeted metabolomics does not allow for the discovery of molecules that are not in the predetermined list [50], whereas untargeted metabolomics does. The molecules detected by metabolomics are metabolites resulting from the action of the microbiota and their quantification provide a clear portrait of the alterations in the metabolic performance of the microbiota under any condition [6]. One study, which used data from a population-based twin study (TwinsUK), estimated stool metabolite associations with adiposity and gut microbial composition. The researchers found that stool metabolites, which were strongly associated with obesity, explained nearly 68% of the variance in gut microbial composition [51]. Thus, understanding how microbiome–metabolome associations relate not only to obesity but also to other chronic disease risk factors could shed light on disease etiology and identify targets for intervention [10].

## 5. Conclusions

In conclusion, 23 observational studies with a wealth of data on dietary intake, microbiome, biomarkers, and health outcomes were identified within KP-INTIMIC for future combined analysis. Crucially, due to common data sharing restrictions, a federated approach similar to the one proposed here appears to be a necessity to make full use of this body of knowledge. The identified studies may promote collaboration initiatives using FAIR data to conduct secondary data analysis that may shed light on the understanding of the role of the human gut microbiome in health and disease, as well as the effects of diet on the composition of the gastrointestinal ecosystem in early infancy, ageing, and in health and disease both within and outside the KP-INTIMIC consortium.

## Figures and Tables

**Table 1:** Characteristics of the identified observational studies within the KP-INTIMIC consortium.

Study [Ref.]	Country	Study Design	Number of Participants [n (M/F)]	Recruitment Years	Population [Age at Recruitment]
DONALD ^1^ study [27]	Germany	Cohort	10,172 (4960/5212)	1985-current (open)	Population-based Convenient sampling (3–6 month)
EPIC-Potsdam [28]	Germany	Cohort	27,548 (10,904/16,644)	1994–1998	Population-based (35–65 year)
LISA ^2^ [29]	Germany	Cohort	3094 (1584/1510)	1997–1999	Population-based (0 year)
NAKO ^3^ [30]	Germany	Cohort	205,184 (101,658/103,52) (Germany)	2014–2019 (baseline)	Population-based (20–69 year)
Diet4MicroGut [31]	Italy	Cohort	153 (64/89)	2012–2013	Population-based (18–60 year)
DORIAN-PISAC ^4^ [32]	Italy	Cohort	90 parent-children trios ([31] 48/42 children)	2011–2014	Population-based (0 year)
Infant microbiome studies (full term, moderately preterm and preterm infants with VLBW ^5^) [33]	Italy	Cohort	87 (N.A.)	2013–2015	Population-based (1–20 days)
Italian Elderly Cohort [34]	Italy	Cohort	201 (101/100)	2012–2015	Population-based (65–79 year)
Octopus (PLIC ^8^)	Italy	Cohort	93 (N.A.)	2021-current	PLIC ^8^: Population-based (N.A.)
IDEFICS/I.Family cohort [35]	Multinational (Cyprus, Estonia, Germany, Hungary, Sweden)	Cohort	IDEFICS baseline: *n* = 16,229; 2-y FU: *n* = 13,596; 6-y FU (I.Family): *n* = 9617 (≈50%/≈50%)	2007–2014 (ongoing: web-based follow-up)	Population-based (2–9.9 (in 2007/2008, IDEFICS children); 2–18 (for siblings of IDEFICS; children recruited in 2013/2014 in the I.Family study))
TransMic [36]	Multinational (Italy and Burkina Faso)	Cohort	300 (N.A.)	2018–2020	Population-based (1–50 year)
LucKI Gut Study [37]	Netherlands	Cohort	107 (63/44)	2017-current	Population-based (0 year)
EarlyMicroHealth [38]	Spain	Cohort	151 (84/67)	2015/2020	Population-based (0–24 month)
ErNst	Germany	Cross-sectional	106 (53/53)	2018	Population-based (20–79 year)
Elderly microbiome studies (centenarians & semi-supercentenarians) [39]	Italy	Cross-sectional	54 (15/39)	2007–2015	Population-based (65–109 year)
DIMISA [40]	Spain	Cross-sectional	184 (52/132)	2012–2015	Population-based Recruitment in wild-living population and elderly homes (19–95 year)
EDILS ^6^ [41]	France	Cohort	280 (47/233)	2015–2021	Disease-based (eating disorders) Recruitment through clinics and through advertisement (for controls) (18+ year)
Medika Study [42]	Italy	Cohort	60 (49/11)	2015–2019	Disease-based (CKD ^7^) Patients from the nephrology, dialysis, and transplantation section of the hospital (56–80 year)
IBD ^9^ South Limburg Cohort [43]	Netherlands	Cohort	4000 (1700 in biobank) (50–55% UC ^10^, 40–45% CD ^11^, & 2–3% IBD ^9^-U)	1991-current	Disease-based (UC ^10^, CD ^11^, IBD ^9^) recruitment through outpatient department & advertisements (for controls) (18+ year)
Maastricht IBS ^12^ Study [44]	Netherlands	Cohort	627 (214/413)	2008-current	Disease-based (IBS ^12^) recruitment through primary, secondary, tertiary care & advertisements (for controls) (18+ year)
Effect of fibers on gut microbiota and SCFA ^13^ in Parkinson patients and healthy references	Belgium	Cross-sectional	63 (N.A.)	2018–2019	Disease-based (Parkinson) Convenience sampling (healthy controls) (55+ year)
FoCus ^14^ [45]	Germany	Cross-sectional	1811 (1133/678)	2014–2015 (Baseline)	Disease- based (Obesity) & Population-based (for controls) Recruitment through population office (1309) and obesity clinic (502) (18–83 year)
MetaCardis [46]	Multinational (Germany, France and Denmark)	Cross-sectional	2189 (1101/1088)	2013–2015	Disease-based (CMD ^15^) Recruitment through clinics and through advertisement (for controls) (18–75 year)

^1^ DONALD: DOrtmund Nutritional and Anthropometric Longitudinally Designed; ^2^ LISA: Influences of Lifestyle-Related Factors on the Human Immune System and Development of Allergies in Children; ^3^ NAKO: German National cohort; ^4^ DORIAN-PISAC: Developmental ORigins of healthy and unhealthy AgeiNg: The Role of Maternal Obesity-Pisa Birth Cohort; ^5^ VLBW: very low birth weight; ^6^ EDILS: Eating Disorders Inventory and Longitudinal Survey; ^7^ CKD: chronic kidney disease; ^8^ PLIC: Progression of Lesions in the Intima of the Carotid; ^9^ IBD: inflammatory bowel disease; ^10^ UC: Ulcerative Colitis; ^11^ CD: Crohn’s disease; ^12^ IBS: irritable bowel syndrome; ^13^ SCFA: short chain fatty acids; ^14^ FoCus: Food Chain Plus; ^15^ CMD: cardiometabolic disease. N.A.: not available.

**Table 2:** Methods used for dietary assessments in the identified observational studies participating in the KP-INTIMIC consortium.

Study	Country	Dietary Intake	24-h Recall	FFQ ^1^	Food Records	Other
DONALD study	Germany	√	—	—	√	—
EPIC-Potsdam	Germany	√	√ M ^2^	√ SQ ^3^	—	—
LISA	Germany	√	—	√ SQ ^3^	—	—
NAKO	Germany	√	√ M ^2^	√ SQ ^3^	—	—
Diet4MicroGut	Italy	√	—	—	√	—
DORIAN-PISAC	Italy	√	√ M ^2^	√ SQ ^3^	—	—
Infant microbiome studies	Italy	√	—	—	—	Interview or visits ^4^
Italian Elderly Cohort	Italy	√	—	—	√	—
Octopus	Italy	√	—	—	—	Questionnaires
IDEFICS/I.Family cohort	Multinational	√	√ M ^2^	√ QU ^5^	—	—
TransMic	Multinational	√	—	√ SQ ^3^		—
LucKI Gut Study	Netherlands	√	—	√ SQ ^3^	—	—
EarlyMicroHealth	Spain	√	—	√ SQ ^3^	—	—
ErNst	Germany	√	—	√ SQ ^3^	—	—
Elderly microbiome studies	Italy	√	—	—	√^6^	—
DIMISA	Spain	√	—	√ SQ ^3^	—	—
EDILS	France	√	—	—	—	Interview
Medika Study	Italy	√	—	—	—	√
IBD South Limburg Cohort	Netherlands	√^6^	—	√ SQ ^3,6^	—	—
Maastricht IBS Study	Netherlands	√	—	√ SQ ^3^	—	—
Effect of fibers on gut microbiota and SCFA in Parkinson patients and healthy references	Belgium	—	—	—	—	—
FoCus	Germany	√	—	√ SQ ^3^	—	—
MetaCardis	Multinational	√	√ M ^2,6^	√ SQ ^3^	—	—

^1^ FFQ: food frequency questionnaire; ^2^ M: multiple; ^3^ SQ: semiquantitative; ^4^ The following information is available: feeding with human (mother’s or donor’s) milk, formula, or mixed; ^5^ QU: qualitative; ^6^ available for a subset of participants.

**Table 3:** Microbiome data available in the observational studies participating in the KP-INTIMIC consortium.

Study	Country	Gut Microbiome		Other Microbiome	
		Stool Sample Collected	Gut Microbiome Measured	Other Samples Collected	Other Microbiome Measured
DONALD study	Germany	√	√	—	—
EPIC-Potsdam	Germany	√	√	—	—
LISA	Germany	√	√	—	—
NAKO	Germany	√	—	Saliva	—
Diet4MicroGut	Italy	√	√	Saliva	√
DORIAN-PISAC	Italy	√	√	—	—
Infant microbiome studies	Italy	√	√	Oral swabs	√
Italian Elderly Cohort	Italy	√	√	—	—
Octopus	Italy	√	√	—	—
IDEFICS/I.Family cohort	Multinational	√	√	—	—
TransMic	Multinational	√	√	—	—
LucKI Gut Study	Netherlands	√	√	—	—
EarlyMicroHealth	Spain	√	√	—	—
ErNst	Germany	√	√	—	—
Elderly microbiome studies	Italy	√	√	—	—
DIMISA	Spain	√	√	—	—
EDILS	France	√	√	—	—
Medika Study	Italy	√	√	—	—
IBD South Limburg Cohort	Netherlands	√	√	—	—
Maastricht IBS Study	Netherlands	√	√	—	—
Effect of fibers on gut microbiota and SCFA in Parkinson patients and healthy references	Belgium	√	√	—	—
FoCus	Germany	√	√	—	—
MetaCardis	Multinational	√	√	—	—

**Table 4:** Gut microbiome assessment in stool samples from the observational studies participating in the KP-INTIMIC consortium.

Study Name	Country	Gut Microbiome			
		Number of Participants with Samples	Method ^1^	Sequencing Platform	Info on Type of Meal Last Eaten Prior Sample Extraction
DONALD study	Germany	128	16S (V3–V4)	Illumina MiSeq	No
EPIC-Potsdam	Germany	3299	16S (V3–V4)	Illumina MiSeq	No
LISA	Germany	166 ^2^	16S (V3–V4)	Illumina MiSeq	No
NAKO	Germany	76,000	—	—	No
Diet4MicroGut	Italy	153	16S (V1–V3)	454 Junior (16S seq), Illumina NextSeq (shotgun)	No
DORIAN-PISAC	Italy	30–80 ^3^	16S (V3–V4)	Illumina MiSeq	No
Infant microbiome studies	Italy	87	16S (V3–V4)	Illumina MiSeq	No
Italian Elderly Cohort	Italy	201	16S (V3–V4)	Illumina MiSeq	No
Octopus	Italy	93	16S (V3–V4)	Illumina MiSeq	No
IDEFICS/I.Family cohort	Multinational	140	16S (V3–V4)	Illumina MiSeq	Yes
TransMic	Multinational	—	Shotgun ITS1-4 ^4^	Illumina NovaSeq Illumina MiSeq	No
LucKI Gut Study	Netherlands	898	16S (V3–V4)	Illumina MiSeq	No
EarlyMicroHealth	Spain	900	16S (V3), shotgun, qPCR	Illumina MiSeq (16S and shotgun)	No
ErNst	Germany	212	16S (V4)	Illumina MiSeq	No
Elderly microbiome studies	Italy	54; 51	16S (V3–V4), shotgun	Illumina MiSeq; Illumina NextSeq (shotgun)	No
DIMISA	Spain	184	qPCR	—	No
EDILS	France	280 ^5^	16S (V5–V6)	Illumina MISeq	No
Medika Study	Italy	27	16S (V1–V3)	Illumina MiSeq	No
IBD South Limburg Cohort	Netherlands	114	16S (V4), shotgun	Illumina MiSeq (16S seq), Illumina HiSeq (shotgun)	—
Maastricht IBS Study	Netherlands	181	16S (V4), shotgun	Illumina MiSeq (16S seq), Illumina HiSeq (shotgun)	—
Effect of fibers on gut microbiota and SCFA in Parkinson patients and healthy references	Belgium	—	16S and culture	—	No
FoCus	Germany	1545	16S (V1–V2 & V3–V4)	Illumina MiSeq	No
MetaCardis	Multinational	2189	shotgun	Ion-proton	No

^1^ 16S: 16S rRNA gene amplicon sequencing (amplified variable regions), shotgun: shotgun metagenomic sequencing; ^2^ in a subset at 6 years; ^3^ depending on the follow-up; ^4^ ITS: internal transcribed spacer; ^5^ at 0-, 18-, and 36-month follow-ups. NR: not reported.

## Data Availability

We exclude this statement.

## Supplementary Materials

The following are available online at https://www.mdpi.com/article/10.3390/nu13093292/s1, Table S1: Description of other measurements assessed in the observational studies participating in KP-INTIMIC, Table S2: Description of biomarkers and metabolome assessed in the observational studies participating in KP-INTIMIC, Table S3: Availability of observational studies to store and share data.
