# Supplementary material for: Identification and Characterization of Human Observational Studies in Nutritional Epidemiology on Gut Microbiomics for Joint Data Analysis
Source: Nutrients. 2021 Sep 21;13(9):3292. doi: 10.3390/nu13093292 (PMC8466729; doi:10.3390/nu13093292)
Supplement: Supplementary file 1 [file nutrients-13-03292-s001.zip › nutrients-1330445-supplementary.pdf]

Supplement Material

Identification and Characterization of Human Observational Studies in Nutritional Epidemiology on Gut Microbiomics for Joint Data Analysis

Mariona Pinart [1], Katharina Nimptsch [1*], Sofia K. Forslund [2,3,4,5,6,7], Kristina Schlicht [8], Miguel Gueimonde [9,10], Patrizia Brigidi [11], Silvia Turroni [12], Wolfgang Ahrens [13,14], Antje Hebestreit [13], Maike Wolters [13], Andreas Dötsch [15], Ute Nöthlings [16], Kolade Oluwagbemigun [16], Rafael R. C. Cuadrat[17], Matthias B. Schulze [17,18,19], Marie Standl [20], Michael Schloter [21], Maria De Angelis [22], Patricia Iozzo [23], Maria Angela Guzzardi [23], Geertrui Vlaemynck [24], John Penders [25], Daisy MAE Jonkers [26], Maya Stemmer [27], Giulia Chiesa [28], Duccio Cavalieri [29], Carlotta De Filippo[30], Danilo Ercolini [31,32], Francesca De Filippis [31,32], David Ribet [33], Najate Achamrah [33,34], Marie-Pierre Tavolacci [33,35], Pierre Déchelotte [33,34], Jildau Bouwman [36], Matthias Laudes [8], and Tobias Pischon [1,3,5,37,38]

[1] Molecular Epidemiology Research Group, Max Delbrück Center for Molecular Medicine in the Helmholtz Association (MDC), 13125 Berlin, Germany
[2] Experimental and Clinical Research Center, A Cooperation of Charité-Universitätsmedizin Berlin and Max Delbrück Center for Molecular Medicine, Lindenberger Weg 80, 13125 Berlin, Germany
[3] Charité-Universitätsmedizin Berlin, Corporate Member of Freie Universität Berlin, Humboldt-Universität zu Berlin, and Berlin Institute of Health, 10117 Berlin, Germany
[4] Max Delbrück Center for Molecular Medicine in the Helmholtz Association, 13125 Berlin, Germany
[5] German Centre for Cardiovascular Research (DZHK), Partner Site Berlin, 10785 Berlin, Germany
[6] Berlin Institute of Health (BIH), 10178 Berlin, Germany
[7] Structural and Computational Biology Unit, European Molecular Biology Laboratory, 69117 Heidelberg, Germany
[8] Institute of Diabetes and Clinical Metabolic Research, University of Kiel, 24105 Kiel, Germany
[9] Department of Microbiology and Biochemistry of Dairy Products, IPLA-CSIC, 33300 Villaviciosa, Spain; mgueimonde@ipla.csic.es
[10] Diet, Microbiota and Health Group, Instituto de Investigación Sanitaria del Principado de Asturias (ISPA), 33011 Oviedo, Spain
[11] Department of Medical and Surgical Sciences, University of Bologna, Via Massarenti 9, 40138 Bologna, Italy
[12] Department of Pharmacy and Biotechnology, University of Bologna, Via Belmeloro 6, 40126 Bologna, Italy
[13] Leibniz Institute for Prevention Research and Epidemiology-BIPS, 28359 Bremen, Germany
[14] Institute of Statistics, Bremen University, 28359 Bremen, Germany
[15] Department of Physiology and Biochemistry of Nutrition, Max Rubner-Institut (MRI)—Federal Research Institute of Nutrition and Food, 76131 Karlsruhe, Germany
[16] Department of Epidemiology, Institute of Nutrition and Food Sciences, University of Bonn, 53115 Bonn, Germany
[17] Department of Molecular Epidemiology, German Institute of Human Nutrition Potsdam-Rehbruecke, 14558 Nuthetal, Germany
[18] Institute of Nutritional Science, University of Potsdam, 14558 Potsdam, Germany
[19] German Center for Diabetes Research (DZD), 85764 München-Neuherberg, Germany
[20] Institute of Epidemiology, Helmholtz Zentrum München–German Research Center for Environmental Health, 85764 Neuherberg, Germany
[21] Research Unit for Comparative Microbiome Analysis, Helmholtz Zentrum München–German Research Center for Environmental Health, 85764 Neuherberg, Germany
[22] Department of Soil, Plant and Food Sciences, University of Bari Aldo Moro, 70126 Bari, Italy
[23] Institute of Clinical Physiology, National Research Council, via Moruzzi 1, 56124 Pisa, Italy
[24] Department Technology and Food, Flanders Research Institute for Agriculture, Fisheries and Food, 9090 Melle, Belgium
[25] Department of Medical Microbiology, NUTRIM School of Nutrition and Translational Research in Metabolism and Care and Public Health Research Institute (CAPHRI), Maastricht University Medical Center+, P.O.B. 616 6200 Maastricht, The Netherlands
[26] Department of Internal Medicine, Division Gastroenterology-Hepatology, NUTRIM School of Nutrition and Translational Research in Metabolism, Maastricht University Medical Center+, P.O.B. 616 6200 Maastricht, The Netherlands
[27] Department of Industrial Engineering and Management, Ben-Gurion University of the Negev, P.O.B. 653 Beer-Sheva, Israel
[28] Department of Pharmacological and Biomolecular Sciences, Università degli Studi di Milano, 20133 Milan, Italy
[29] Department of Biology, University of Florence, Via Madonna del Piano 6, 50019 Sesto Fiorentino, Florence, Italy
[30] Institute of Agricultural Biology and Biotechnology National Research Council Via Moruzzi, 1, 56124 Pisa, Italy
[31] Department of Agricultural Sciences, University of Naples Federico II, 80055 Portici, Italy
[32] Task Force On Microbiome Studies, University of Naples Federico II, 80134 Naples, Italy
[33] INSERM UMR 1073 "Nutrition, Inflammation and Gut-brain axis dysfunctions", UNIROUEN, Normandie University, 76000 Rouen, France
[34] Department of Nutrition, CHU Rouen, 76000 Rouen, France

[35] INSERM CIC-CRB 1404, CHU Rouen, 76000 Rouen, France
[36] Microbiology and Systems Biology Group, TNO, Utrechtseweg 48, 3704HE Zeist, The Netherlands
[37] Biobank Technology Platform, Max Delbrück Center for Molecular Medicine in the Helmholtz Association (MDC), 13125 Berlin, Germany
[38] Biobank Core Facility, Berlin Institute of Health at Charité—Universitätsmedizin Berlin, 10178 Berlin, Germany

Corresponding author:
Katharina Nimptsch; E-Mail: Katharina.Nimptsch@mdc-berlin.de; Tel.: +49 30 9046 4573

Supplement Table S1. Description of other measurements assessed in the observational studies participating in KP-INTIMIC.

Study	Country	Alcohol consumption	Tobacco consumption	PA[1]		Sedentary behaviour	Anthropometry		SES[4]	Medication	Health status
				Q[2]	M[3]		Q[2]	M[3]			
DONALD study	Germany	√	√	√	—	—	—	√	√	√	√
EPIC-Potsdam	Germany	√	√	√	√	√	—	√	√	√	√
LISA	Germany	√	√	√	—	√	√	√	√	√	√
NAKO	Germany	√	√	√	√	—	—	√	√	√	√
Diet4MicroGut	Italy	√	√	—	—	√	√	—	√	√	√
DORIAN-PISAC	Italy	√	√	√	—	—	—	√	√	√	√
Infant microbiome studies	Italy	—	—	—	—	—	—	√[5]	√[5]	√	√
Italian Elderly Cohort	Italy	√	—	√	—	√	—	√	√[5]	√	√
Octopus	Italy	√	√	√	—	√	—	√	√	√	√
IDEFICS/ I.Family cohort	Multinational	√	√	√	√	√	—	√	√	√	√
TransMic	Multinational	√	√	√	—	√	√	√	√	√	√
LucKI Gut Study	Netherlands	√	√	√	—	—	√	√	√	√	√
EarlyMicroHealth	Spain	—	—	—	—	—	—	√	√	√	√
ErNst	Germany	√	√	—	√	—	—	√	√	√	√
Elderly microbiome studies	Italy	√[5]	√[5]	√[5]	—	√[5]	—	√[5]	√[5]	√	√
DIMISA	Spain	√	√	√	—	√	—	√	√	√	√
EDILS	France	√	√	—	—	—	—	√	√	√	√
Medika Study	Italy	—	—	—	—	—	—	√	√	√	√
IBD South Limburg Cohort	Netherlands	√[5]	√	—	—	—	—	√[5]	—	√	√
Maastricht IBS Study	Netherlands	√	√	—	—	—	√	—	√	√	√
Effect of fibers on gut microbiota and SCFA in Parkinson patients and healthy references	Belgium	—	—	—	—	—	√		√	√	√
FoCus	Germany	√	√	√	—	√	√	√	√	√	√
MetaCardis	Multinational	√	√	√	—	√	—	√	√	√	√

[1] PA: physical activity; [2] Q: questionnaire-based; [3] M: measured (objective measurement); [4] SES: socioeconomic status; [5] data available in a subset of participants.

Supplement Table S2. Description of biomarkers and metabolome assessed in the observational studies participating in KP-INTIMIC.

Study	Country	Blood lipids	Glucose/Insulin	Inflammatory markers	Adiposity markers	Metabolome
DONALD study	Germany	√	√	√	√	√ (urine/plasma)
EPIC-Potsdam	Germany	√	√	√	√	—
LISA	Germany	√	√	√	√	√ (serum)
NAKO	Germany	√	√	—	—	—
Diet4MicroGut	Italy	—	—	—	—	√ (urine/stool)
DORIAN-PISAC	Italy	√	√	√	√	√ (urine/stool)
Infant microbiome studies	Italy	—	—	—	—	—
Italian Elderly Cohort	Italy	√	√	√	√	√ (serum)
Octopus	Italy	√	√	√	—	√ (plasma/stool)
IDEFICS/ I.Family cohort	Multinational	√	√	√	√	√ (urine/serum/stool))
TransMic	Multinational	√	√	√	—	√ (stool)
LucKI Gut Study	Netherlands	—	—	—	—	—
EarlyMicroHealth	Spain	—	—	—	—	√ (stool)
ErNst	Germany	—	—	—	—	—
Elderly microbiome studies	Italy	—	—	√	—	√ (urine/serum)
DIMISA	Spain	√	√	√	√	—
EDILS	France	√	√	√	√	—
Medika Study	Italy	√	√	√	—	√ (urine)
IBD South Limburg Cohort	Netherlands	—	—	√	—	—
Maastricht IBS Study	Netherlands	—	—	√	—	√ (stool)
Effect of fibers on gut microbiota and SCFA in Parkinson patients and healthy references	Belgium	—	—	—	—	√ (stool)
FoCus	Germany	√	√	√	√	√ (urine/serum)
MetaCardis	Multinational	√	√	√	√	√ (urine/serum)

Supplement Table S3. Availability of observational studies to store and share data.

Study	International/ National/ Regional	EC approval	IC provided	Agreed to store and share raw data		Willing to store and share metadata for federated joint analysis
				within INTIMIC	Other projects	
DONALD study	Germany	Yes	Yes	Yes	Yes, with permission from PI[1]	Yes, with permission from PI[1]
EPIC-Potsdam	Germany	Yes	Yes	No	No	Yes
LISA	Germany	Yes	Yes	No	requires cooperation contract and MDTA[2] and approval from SC[3]	requires cooperation contract and MDTA[2] and approval from SC[3]
NAKO	Germany	Yes	Yes	Requires approval from the NAKO eV	Requires approval from the NAKO eV	Requires approval from the NAKO eV
Diet4MicroGut	Italy	Yes	Yes	Yes	Yes	Yes
DORIAN-PISAC	Italy	Yes	Yes	No	depends on kind of data, permission of scientists involved, and anonymized	depends on kind of data, permission of scientists involved, and anonymized
Infant microbiome studies	Italy	Yes	Yes	Yes (microbiome data)	Yes (microbiome data)	Yes, after obtaining consent of the partners involved in the nutritional/health topics
Italian Elderly Cohort	Italy	Yes	Yes	Yes (microbiome data)	Yes (microbiome data)	Yes, after obtaining consent of the partners involved in the nutritional/health topics
Octopus	Italy	Yes	Yes	with permission from Octopus consortium	with permission from Octopus consortium	with permission from Octopus consortium
IDEFICS/ I.Family cohort	Germany	Yes	Yes	Yes, after obtaining consent of the PI[1]	Yes, after obtaining consent of the PI[1]	Yes, after obtaining consent of the PI[1]
TransMic	Multinational	Yes	Yes	Not yet	No	with permission from TransMic consortium
LucKI Gut Study	Netherlands	Yes	Yes	No	Yes, with permission from SC[3], anonymized and with MDTA[2]	Yes, with permission from SC[3], anonymized and with MDTA[2]
EarlyMicroHealth	Spain	Yes	Yes	Yes	Yes	Yes
ErNst	Germany	Yes	Yes	Yes	Yes	Yes
Elderly microbiome studies	Italy	Yes	Yes	Yes (microbiome data)	Yes (microbiome data)	Yes, after obtaining consent of the partners involved in the nutritional/health topics
DIMISA	Spain	Yes	Yes	Yes	Yes	Yes
EDILS	France	Yes	Yes	No	Yes, with permission	Yes if anonymized
Medika Study	Italy	Yes	Yes	Yes	Yes	Yes
IBD South Limburg Cohort	Netherlands	Yes	Yes	No	Yes, with permission from SC[3], anonymized and with MDTA[2]	Yes, with permission from SC[3], anonymized and with MDTA[2]

Maastricht IBS Study	Netherlands	Yes	Yes	No	Yes, with permission from SC[3], anonymized and with MDTA[2]	Yes, with permission from SC[3], anonymized and with MDTA[2]
Effect of fibers on gut microbiota and SCFA in Parkinson patients and healthy references	Belgium	Yes	Yes	No	No	No
FoCus	Germany	Yes	Yes	No	Yes, with permission from P2N biobank and anonymized	Yes, with permission from P2N biobank and anonymized
MetaCardis	Multinational	Yes	Yes	No	with permission from SC[3] and anonymized	Yes, with permission from SC[3] and anonymized

[1]PI: Principal Investigator; [2]MDTA: Material and data Transfer agreement; [3] SC: Steering Committee
